# Medicated Seton and Fistulotomy or Fistulectomy in the Management of Simple Anal Fistula: A Prospective Comparative Study

**DOI:** 10.7759/cureus.100240

**Published:** 2025-12-28

**Authors:** Pallav Sachar, Rajan Sood, Deepak Kumar, Upender K Chandel, Samir Anand

**Affiliations:** 1 Department of General Surgery, Maharishi Markandeshwar Medical College and Hospital, Solan, IND

**Keywords:** fistula in ano, fistulectomy, fistulotomy, medicated seton, recurrence, sphincter preservation

## Abstract

Background

Fistula in ano is a persistent anorectal condition associated with pain, discharge, and recurrence. Traditional procedures such as fistulotomy and fistulectomy remain the standard of care for simple fistulae but may be associated with postoperative discomfort, delayed healing, and potential sphincter injury. Medicated seton offers a sphincter-preserving alternative that may enhance healing and reduce morbidity. The present study aims to compare the clinical effectiveness and safety of medicated seton versus fistulotomy or fistulectomy in the treatment of simple anal fistula.

Methods

This prospective comparative study enrolled 50 patients with simple anal fistula (St. James Grade I-II) between January 2024 and June 2025. In accordance with American Society of Colon and Rectal Surgeons (ASCRS) guidelines, only simple low intersphincteric and low trans-sphincteric fistulas were included. Patients were allocated into two groups of 25 each: medicated seton or fistulotomy/fistulectomy. Parameters assessed included operative time, postoperative pain (visual analog scale (VAS) at six hours), hospital stay, complications, work-off period, healing time, recurrence, patient satisfaction, and overall efficacy. Data were analyzed using the t-test and chi-square test, with p < 0.05 considered significant.

Results

Baseline demographics were comparable between groups. Operative time was significantly lower in the medicated seton group (18.6 ± 3.2 minutes) compared with the fistulotomy/fistulectomy group (28.4 ± 4.5 minutes; p < 0.001). Hospital stay (2.3 ± 0.8 vs 4.1 ± 1.2 days; p < 0.001) and postoperative pain scores (3.1 ± 1.2 vs 6.4 ± 1.6; p < 0.001) were also significantly reduced with medicated seton. Postoperative complications were low in both groups, with wound infection occurring in 2 (8%) vs 6 (24%), bleeding in 1 (4%) vs 3 (12%), and flatus incontinence in 0 vs 2 (8%) patients, respectively. Recovery outcomes favored medicated seton, including a shorter work-off period (8.2 ± 2.4 vs 15.6 ± 3.7 days; p < 0.001) and faster healing (6.2 ± 1.5 vs 8.9 ± 1.8 weeks; p < 0.001). Recurrence at three months was low in both groups, occurring in 1 (4%) vs 3 (12%) patients (p < 0.602). Overall efficacy was achieved in 23 (92%) patients in the medicated seton group compared with 19 (78%) in the fistulotomy/fistulectomy group.

Conclusion

Medicated seton was associated with reduced operative time, decreased postoperative pain, shorter hospitalization, quicker return to routine activities, and favorable healing outcomes. It offers a safe, effective, and sphincter-preserving option for managing simple anal fistula, with comparable recurrence rates to traditional surgery.

## Introduction

A fistula is an abnormal connection that forms either between two internal organs or between an internal organ and the skin. This usually develops as a result of infection, trauma, or other disease processes. Fistula in ano refers to an abnormal tract extending from the anal canal or rectum to the perianal surface [1]. The condition has been recognized since ancient times, with one of the earliest descriptions attributed to Hippocrates around 430 B.C., who described surgical management and introduced both fistulectomy and the use of a seton fashioned from horsehair wrapped with lint threads [2].

Recurrence of fistula in ano is frequently observed in clinical practice, often due to incomplete or unsuccessful prior interventions. Standard surgical treatments may be associated with postoperative concerns such as sphincter incontinence, anal stenosis, and recurrence [3]. These procedures may also require prolonged hospital stays, repeated painful dressings, and significant loss of work days, making them less favorable as primary treatment options for fistula in ano [4]. Achieving reliable healing while preserving sphincter function remains a major challenge for the surgeon.

The medicated seton technique, initially described by the ancient Indian surgeon Sushruta, has regained importance as a viable therapeutic approach [5]. For straightforward and low-lying fistulae, conventional surgery remains effective, but in more complex cases where a larger portion of the sphincter is involved, there is considerable concern regarding functional impairment with traditional procedures [6]. As a result, several sphincter-preserving techniques have been introduced with the intent of reducing sphincter injury while maintaining adequate healing outcomes [7].

Setons have been used since antiquity, and contemporary medicated setons have demonstrated a high rate of success, with reports indicating cure rates exceeding 98% [8]. This method can often be performed on an outpatient basis, avoiding the need for prolonged hospitalization or advanced operating room facilities. Patients typically remain mobile and able to continue routine activities throughout treatment, making the technique especially suitable for primary care and rural healthcare settings [8].

The objective of this study is to compare the management of fistula in ano using medicated seton versus fistulectomy, with specific attention to healing outcomes, postoperative complications, and recurrence.

## Materials and methods

Study design and duration

This study was conducted as a prospective comparative study to compare the efficacy and outcomes of medicated seton placement versus fistulotomy/fistulectomy in the management of simple anal fistulas. The seton consisted of a linen/cotton thread coated with herbal/alkaline medicaments (kshar, turmeric, ghee) prepared per classical methods. The medicated coating promotes controlled chemical debridement and fibrosis. The trial was carried out over an 18-month period at a tertiary care surgical center between January 2024 and June 2025. Patients with simple anal fistula (St. James Grade I-II) were enrolled. In accordance with American Society of Colon and Rectal Surgeons (ASCRS) guidelines, only simple low intersphincteric and low trans-sphincteric fistulas were included. A total of 50 eligible patients were enrolled and allocated alternately into two equal groups of 25 patients each using a non-randomized convenience allocation method after confirmation of eligibility based on clinical examination, MRI findings, and fistula classification. This study was reported in accordance with the Strengthening the Reporting of Observational Studies in Epidemiology (STROBE) guidelines for observational research.

Inclusion and exclusion criteria

Patients presenting with suspected anal fistula were first evaluated clinically, followed by an MRI fistulogram to confirm tract anatomy. Only simple fistulas corresponding to St. James Grade I-II [9] and ASCRS-defined simple low intersphincteric or low trans-sphincteric types were included. Inclusion criteria were: age 18-65 years, single tract, single external opening, and absence of acute abscess. Exclusion criteria included Crohn's disease, tuberculosis, HIV, pregnancy, recurrent or complex fistulas, multiple external openings, and immunocompromised states.

Surgical procedures

After confirming eligibility and securing informed consent, patients were divided into two intervention arms using alternate allocation. All surgeries were performed under saddle block anesthesia. In the medicated seton group, the fistula tract was carefully delineated with a malleable probe, following which a medicated seton thread was passed through the tract and secured externally. Patients were reviewed weekly, and the seton was gradually tightened to allow controlled division of the tract with concurrent fibrosis and healing. In the fistulotomy/fistulectomy group, the tract was similarly probed, and the surgeon elected either to lay open the tract (fistulotomy) or excise it entirely (fistulectomy) based on intraoperative findings. Hemostasis was ensured, and the wound was left to heal by secondary intention following standard proctologic surgical principles. Performance bias was minimized by ensuring that all procedures were performed by surgeons trained in both techniques.

Postoperative care and follow-up

Although medicated seton placement is performed as an outpatient procedure in some centers, in our institution, the procedure is carried out under saddle block anesthesia in the operating room. Patients are therefore kept under short-term inpatient observation for monitoring of anesthesia recovery, postoperative pain control, early assessment of seton tension, and to provide structured guidance on sitz baths and wound care. Standard postoperative care, including analgesics and antibiotics, was provided to patients, and they were advised to perform warm sitz baths twice daily. They were reviewed weekly in the outpatient clinic until complete wound healing was documented. Subsequent follow-up assessments were scheduled at one month and three months post-operatively to monitor recovery and evaluate recurrence. Detection bias was reduced by using predefined outcome assessments and consistent follow-up at scheduled intervals.

Outcome measures

Postoperative pain was assessed at six hours using a 10-cm visual analog scale (VAS), in which patients marked their pain intensity from 0 (no pain) to 10 (worst possible pain) [10]. Scarring was evaluated at three months using a simple clinical grading system classifying postoperative scarring as Grade I (minimal scar without distortion), Grade II (visible fibrotic change without functional impairment), or Grade III (dense fibrotic scar associated with retraction or cosmetic concern) [11]. Patient satisfaction was recorded at follow-up using a three-point Likert scale categorized as excellent, good, or fair, based on the patient’s subjective overall experience with the treatment outcome [12]. VAS, scarring grade, and patient satisfaction scoring tools used in this study are open-access clinical instruments and do not require permission for non-commercial research use. No copyrighted scoring system was utilized. All variables were recorded systematically and analyzed to compare the effectiveness and safety of the two interventions. Wound infection was diagnosed based on predefined clinical criteria consisting of purulent discharge, periwound erythema, local tenderness, or the requirement for antibiotic therapy. No numerical wound infection scoring scale was used. The recurrence was defined as the return of discharge, presence of a new external opening, or radiological evidence of a persistent tract after documented initial healing. Overall efficacy was defined as complete healing with no recurrence or major complications, and patient satisfaction was rated as excellent or good. Although weekly tightening visits are integral to medicated seton therapy, the exact number of tightening sessions was not recorded prospectively. The work-off period reflects the time taken for the patient to resume routine activities and does not separately quantify weekly follow-up attendance.

Sample size and statistical analysis

The sample size was calculated a priori based on the expected difference in mean healing time between the two treatment groups. A clinically meaningful difference of two weeks was assumed, with an estimated standard deviation of 2.5 weeks, power of 80%, and a two-sided alpha level of 0.05. Based on these assumptions, a minimum of 23 patients were required in each group. To account for an anticipated 10% loss to follow-up, the final sample size was rounded to 25 patients per group, resulting in a total study population of 50 patients. Data were analyzed using SPSS version 25 (IBM Corp., Armonk, New York, USA). Normality of continuous data was assessed using the Shapiro-Wilk test prior to statistical comparison. Continuous variables were expressed as mean ± standard deviation (SD) and compared using the independent t-test. Categorical variables were presented as frequencies/percentages and analyzed using Fisher's exact test. A p-value <0.05 was considered statistically significant. There were no missing data in the variables analyzed.

## Results

The baseline demographic and clinical characteristics were comparable between the two groups. The mean age was 39.2 ± 8.4 years in the medicated seton group and 40.1 ± 7.9 years in the fistulotomy/fistulectomy group (t = 0.39; p < 0.698). Gender distribution was similar, with 18 (72%) males and 7 (28%) females in the medicated seton group compared to 17 (68%) males and 8 (32%) females in the surgical group (χ² = 0.09; p < 0.757). The mean duration of symptoms did not differ significantly, measuring 5.6 ± 1.2 months versus 5.8 ± 1.5 months (t = 0.52; p < 0.605). Fistula types were almost equally distributed across groups, with St. James grade I seen in 14 (56%) cases and grade II in 11 (44%) cases in the medicated seton group, compared to 15 (60%) and 10 (40%) cases, respectively, in the fistulotomy/fistulectomy group (χ² = 0.08; p < 0.774) (Table 1).

**Table 1:** Baseline demographic and clinical characteristics comparing medicated seton and fistulotomy/fistulectomy groups.

Parameter	Domain	Medicated seton	Fistulotomy/fistulectomy	Statistics	P-value
Mean age (years)		39.2 ± 8.4	40.1 ± 7.9	t = 0.39	<0.698
Gender	Male	18 (72%)	17 (68%)	Fisher's exact test	<0.757
	Female	7 (28%)	8 (32%)		
Mean symptom duration (months)		5.6 ± 1.2	5.8 ± 1.5	t = 0.52	<0.605
Type of fistula	St. James I	14 (56%)	15 (60%)	Fisher's exact test	<0.774
	St. James II	11 (44%)	10 (40%)		

Operative time was significantly shorter in the medicated seton group, with a mean duration of 18.6 ± 3.2 minutes compared to 28.4 ± 4.5 minutes in the fistulotomy/fistulectomy group (t = 8.87; p < 0.001). The mean duration of hospital stay was likewise reduced in patients treated with medicated seton (2.3 ± 0.8 days) compared to those undergoing fistulotomy/fistulectomy (4.1 ± 1.2 days), demonstrating a statistically significant difference (t = 6.24; p < 0.001) (Table 2).

**Table 2:** Operative and hospital stay outcomes in patients treated with medicated seton versus fistulotomy/fistulectomy. *Statistically significant p-value < 0.001.

Parameter	Medicated seton	95% CI	Fistulotomy/fistulectomy	95% CI	Statistics	P-value
Operative time (min)	18.6 ± 3.2	17.28–19.92	28.4 ± 4.5	26.54–30.26	t = 8.87	<0.001*
Hospital stay (days)	2.3 ± 0.8	1.97–2.63	4.1 ± 1.2	3.60–4.60	t = 6.24	<0.001*

Postoperative pain at six hours was significantly lower in the medicated seton group, with mean VAS scores of 3.1 ± 1.2 compared to 6.4 ± 1.6 in the fistulotomy/fistulectomy group (t = 8.25; p < 0.001). Wound infection occurred in 2 (8%) patients in the medicated seton group and 6 (24%) patients in the surgical group; however, this difference was not statistically significant (p < 0.246). Bleeding was noted in 1 (4%) patient treated with medicated seton compared to 3 (12%) patients undergoing fistulotomy/fistulectomy (p < 0.999). Flatus incontinence was reported in 2 (8%) patients in the surgical group but was absent in the medicated seton group (p < 0.489) (Table 3).

**Table 3:** Postoperative pain and complication profiles in patients undergoing medicated seton versus fistulotomy/fistulectomy. *Statistically significant p-value < 0.001.

Parameter	Medicated seton	95% CI	Fistulotomy/fistulectomy	95% CI	Statistics	P-value
VAS pain (6 h)	3.1 ± 1.2	2.60–3.60	6.4 ± 1.6	5.74–7.06	t = 8.25	<0.001*
Wound infection	2 (8%)	–	6 (24%)	–	Fisher’s exact test	<0.246
Bleeding (cases)	1 (4%)	–	3 (12%)	–	Fisher’s exact test	<0.999
Incontinence (flatus)	0	–	2 (8%)	–	Fisher’s exact test	<0.489

The mean duration of the work-off period was significantly shorter in the medicated seton group (8.2 ± 2.4 days) compared with the fistulotomy/fistulectomy group (15.6 ± 3.7 days), demonstrating a strong statistical difference (t = 8.39; p < 0.001). Recurrence at three months was low in both groups, occurring in 1 (4%) patient in the medicated seton group and 3 (12%) patients in the surgical group, with no statistically significant difference (p < 0.609). Scarring outcomes were comparable across treatment arms: in the medicated seton group, grade I, II, and III scarring were seen in 20 (80%), 4 (16%), and 1 (4%) patients, respectively, whereas in the fistulotomy/fistulectomy group, they were observed in 15 (60%), 6 (24%), and 4 (16%) patients, respectively, with no significant variation between groups (p < 0.269) (Table 4).

**Table 4:** Recovery and follow-up outcomes comparing medicated seton and fistulotomy/fistulectomy groups. *Statistically significant p-value < 0.001.

Parameter	Domain	Medicated seton	95% CI	Fistulotomy/fistulectomy	95% CI	Statistics	P-value
Work-off period (days)	–	8.2 ± 2.4	7.21–9.19	15.6 ± 3.7	14.07–17.13	t = 8.39	<0.001*
Recurrence (three months)	–	1 (4%)	–	3 (12%)	–	Fisher’s exact test	<0.609
Scarring grade	I	20 (80%)	–	15 (60%)	–	Fisher’s exact test	<0.269
	II	4 (16%)	–	6 (24%)	–		
	III	1 (4%)	–	4 (16%)	–		

Mean healing time was significantly shorter in the medicated seton group, measuring 6.2 ± 1.5 weeks compared with 8.9 ± 1.8 weeks in the fistulotomy/fistulectomy group (t = 5.76; p < 0.001). Patient satisfaction ratings showed a greater proportion of excellent outcomes in the medicated seton group, with 20 (80%) reporting “excellent” satisfaction, 4 (16%) reporting “good,” and 1 (4%) reporting “fair,” compared to 14 (56%), 6 (24%), and 5 (20%), respectively, in the surgical group; however, these differences were not statistically significant (p < 0.127). Overall efficacy, defined as complete healing without recurrence or major complications and patient satisfaction rated as “excellent” or “good,” was higher in the medicated seton group, with 23 (92%) achieving favorable outcomes versus 19 (78%) in the fistulotomy/fistulectomy group, and this difference reached statistical significance (p < 0.009) (Table 5).

**Table 5:** Overall efficacy comparison between medicated seton and fistulotomy/fistulectomy groups.

Parameter	Domain	Medicated seton	95% CI	Fistulotomy/fistulectomy	95% CI	Statistics	P-value
Mean healing time (weeks)	–	6.2 ± 1.5	5.58–6.82	8.9 ± 1.8	8.16–9.64	t = 5.76	<0.001
Patient satisfaction	Excellent	20 (80%)	–	14 (56%)	–	Fisher’s exact test	<0.127
	Good	4 (16%)	–	6 (24%)	–		
	Fair	1 (4%)	–	5 (20%)	–		
Overall efficacy (%)	–	23 (92%)	–	19 (78%)	–	Fisher’s exact test	<0.009

**Figure 1:**
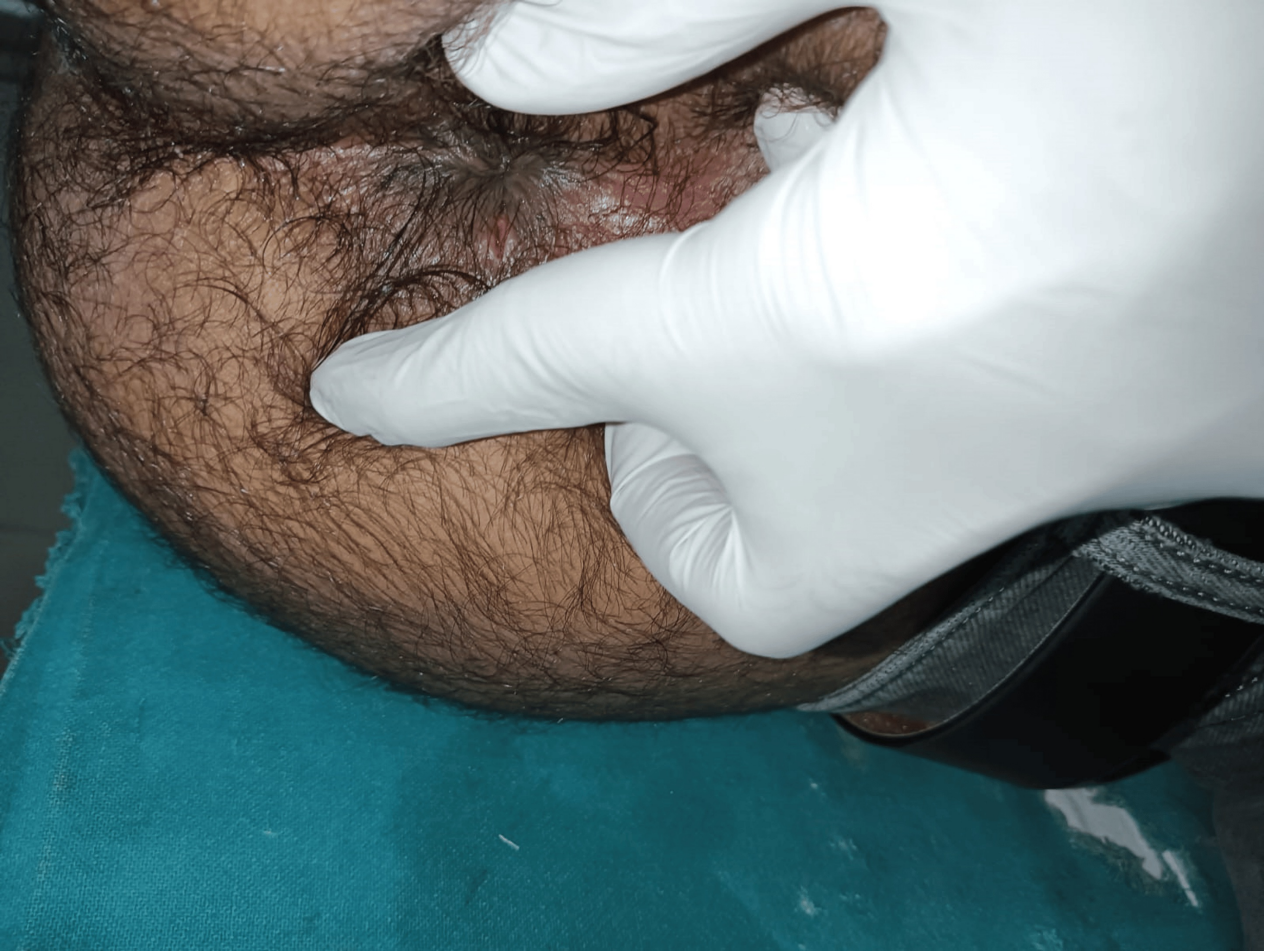
Postoperative wound appearance at three months showing a completely healed tract with healthy surrounding tissue.

Figure 1 demonstrates the postoperative status at three months, showing a well-healed fistula tract with complete epithelialization and no evidence of discharge, induration, or residual opening. The surrounding perianal skin appears healthy, and gentle separation of the gluteal region confirms closure of the previous tract. This indicates successful healing without recurrence or complications at follow-up (Figure 1).

## Discussion

This study shows that medicated seton offers clear clinical advantages over fistulotomy and fistulectomy in the management of simple low anal fistula. Both groups were comparable at baseline, ensuring that differences observed in outcomes were attributable to the intervention itself. Across multiple parameters, medicated seton demonstrated superior perioperative and postoperative performance.

In the present study, operative time was considerably shorter in the medicated seton group (18.6 ± 3.2 minutes) compared with the fistulotomy or fistulectomy group (28.4 ± 4.5 minutes), reflecting a statistically significant difference (t = 8.87, p < 0.001). This trend is consistent with the findings of Madankar et al., who reported that the medicated seton technique required notably less time than the fistulectomy procedure [13]. Similar observations have been documented in the studies conducted by Limura et al., Shukla et al., Reddy et al., and Hiremath [7,8,14,15].

A comparable pattern was observed for the duration of hospital stay in our study, which was significantly shorter in the medicated seton group (2.3 ± 0.8 days) than in the surgical group (4.1 ± 1.2 days) (t = 6.24, p < 0.001). Reddy et al. evaluated 44 patients with fistula in ano and reported that operative time in the medicated seton group ranged from 18.2 to 33.54 minutes, whereas patients undergoing fistulectomy required between 47 and 64.6 minutes [14]. Madankar et al. also noted that patients treated with medicated seton experienced a significantly shorter hospital stay compared with those undergoing conventional fistulectomy [13]. In the study by Khan et al., the mean hospital stay and recovery time were longer in the fistulectomy group [16].

In our study, postoperative pain at six hours was significantly lower in the medicated seton group (3.1 ± 1.2) compared to the fistulotomy/fistulectomy group. In the study conducted by Kumar, patients treated with seton experienced notably lower mean pain scores (3.64) when compared with those who underwent fistulotomy (6.57) and fistulectomy (4.78), with the difference being statistically significant (p < 0.001) [3]. Correspondingly, the work-off period in our study was substantially shorter in the medicated seton group (8.2 ± 2.4 days) compared with the fistulotomy or fistulectomy group (15.6 ± 3.7 days), demonstrating a significant difference (t = 8.39, p < 0.001). Madankar et al. reported similar findings, noting that patients treated with a seton resumed routine activities earlier because they experienced less postoperative pain and did not have an open wound, unlike those who underwent fistulectomy. As a result, many patients were able to return to work as early as the day after the procedure, and the medicated seton group had significantly fewer days off work (p < 0.001) [13]. These observations are consistent with the outcomes described by Limura et al. and Reddy et al., who also documented shorter work-off periods in the seton group when compared with conventional fistulectomy [7,14]. Quinn et al. demonstrated that fistulotomy requires less operative time than fistulectomy because it avoids complete tract excision [17]. Malik and Nelson also highlighted that simpler, tissue-sparing techniques generally reduce operative duration compared with more extensive excisional procedures [2]. In line with these observations, Iqbal et al. noted that sphincter-preserving approaches typically require shorter operative times while minimizing functional risks [6].

In the present study, mean healing time was significantly shorter in the medicated seton group (6.2 ± 1.5 weeks) than in the fistulotomy or fistulectomy group (8.9 ± 1.8 weeks), demonstrating a clear statistical difference (t = 5.76, p < 0.001). In the study conducted by Kumar, the seton group also showed the fastest wound healing, with an average duration of 7.5 days, whereas healing required 8.7 days following fistulectomy and 20.78 days after fistulotomy, again demonstrating a significant difference (p < 0.001) [3]. In contrast, Madankar et al. reported longer treatment duration in the seton group, with a mean healing period of 67.35 days, whereas the fistulectomy group healed in a mean of 24.9 days. Since medicated seton therapy requires a staged approach with weekly visits for seton replacement, the overall time to complete healing was higher in their seton cohort (p = 0.181) [13]. Findings from Limura et al., Shukla et al., and Gupta et al. also support this pattern, showing differences in healing duration across treatment modalities [7,8,18]. The systematic review by Quinn et al. reported that the available evidence did not clearly favor either fistulectomy or fistulotomy with respect to healing time, as no definitive superiority of one technique over the other could be established [17]. Malik and Nelson similarly noted that healing after fistulotomy or fistulectomy may be prolonged due to the larger open wounds created during excisional surgery, highlighting the potential advantages of less invasive, sphincter-preserving methods [2]. In line with this, Iqbal et al. found that sphincter-preserving techniques maintain healing rates comparable to conventional procedures while minimizing tissue trauma [6].

In the present study, recurrence at three months was low in both treatment arms, with one case (4%) in the medicated seton group and three cases (12%) in the surgical group, and this difference was not statistically significant (χ² = 0.27, p < 0.602). Madankar et al., in their study of 46 patients, reported no recurrence following medicated seton therapy, whereas the fistulectomy group demonstrated a 10% recurrence rate. However, because their follow-up ranged from six to eighteen months, an exact recurrence estimate is difficult to determine [13]. Reddy et al., who evaluated 44 cases of fistula in ano, also observed a recurrence rate of 0% with medicated seton compared to 20% following fistulectomy [14]. In the study by Sharma and Ahmad, the seton technique recorded the lowest recurrence rate at 8%, while fistulectomy showed the highest rate at 28%, and this difference was statistically significant (p < 0.000) [19]. Similar observations have been made by Izadpanah et al. [20]. Quinn et al. found no significant difference in recurrence between fistulotomy and fistulectomy for simple anal fistulas, indicating that both procedures are effective when the tract is adequately treated [17]. Malik and Nelson similarly reported that recurrence rates in simple fistulas are generally low across surgical techniques provided the internal opening is properly addressed [2]. Consistent with these observations, Iqbal et al. showed that sphincter-preserving procedures do not increase recurrence risk when executed correctly, supporting the safety of less invasive approaches [6].

The strengths of this study include its prospective comparative design and standardized operative and postoperative protocols, reducing variability in patient management. However, some limitations should be acknowledged. The sample size was relatively small, and a follow-up of three months may not capture late recurrences or long-term functional outcomes. Another limitation of this study is that the exact number of weekly seton-tightening visits was not recorded. Repeated outpatient visits may contribute additional travel time, inconvenience, or work absenteeism for patients, and this potential burden could not be quantified in the present analysis. The findings may not be generalizable to complex or recurrent fistulas since only simple cases were included. The follow-up period of three months was relatively short and may not adequately capture late recurrences, which typically manifest over a longer duration. Preoperative images were not uniformly captured for all patients, limiting direct visual comparison of pre- and postoperative fistula anatomy. Future studies with larger cohorts and extended follow-up are needed to better define long-term recurrence rates, continence status, and overall quality-of-life outcomes.

## Conclusions

This study demonstrates that medicated seton is an effective and well-tolerated option for managing simple anal fistula. Compared with fistulotomy and fistulectomy, medicated seton resulted in shorter operative time, reduced postoperative pain, quicker healing, and faster return to work, while maintaining a low complication profile and preserving sphincter function. Recurrence rates were similarly low in both groups. These findings support medicated seton as a safe, minimally invasive, and patient-friendly alternative for simple fistula management. Larger studies with longer follow-up are recommended to confirm long-term healing, functional outcomes, and long-term recurrence rates.
